# Fractional anisotropy shows differential reduction in frontal-subcortical fiber bundles—A longitudinal MRI study of 76 middle-aged and older adults

**DOI:** 10.3389/fnagi.2015.00081

**Published:** 2015-05-15

**Authors:** Alexandra Vik, Erlend Hodneland, Judit Haász, Martin Ystad, Astri J. Lundervold, Arvid Lundervold

**Affiliations:** ^1^Department of Biological and Medical Psychology, University of BergenBergen, Norway; ^2^Department of Biomedicine, University of BergenBergen, Norway; ^3^Department of Clinical Medicine, University of BergenBergen, Norway; ^4^Kavli Research Center of Aging and Dementia, Haraldsplass Deaconess HospitalBergen, Norway; ^5^Department of Radiology, Haukeland University HospitalBergen, Norway

**Keywords:** diffusion weighted imaging, 3D MRI, fractional anisotropy, tractography, tract parametrization, healthy aging, longitudinal study, frontal-subcortical fibers

## Abstract

Motivated by the frontal- and white matter (WM) retrogenesis hypotheses and the assumptions that fronto-striatal circuits are especially vulnerable in normal aging, the goal of the present study was to identify fiber bundles connecting subcortical nuclei and frontal areas and obtain site-specific information about age related fractional anisotropy (FA) changes. Multimodal magnetic resonance image acquisitions [3D T1-weighted and diffusion weighted imaging (DWI)] were obtained from healthy older adults (*N* = 76, range 49–80 years at inclusion) at two time points, 3 years apart. A subset of the participants (*N* = 24) was included at a third time-point. In addition to the frontal-subcortical fibers, the anterior callosal fiber (ACF) and the corticospinal tract (CST) was investigated by its mean FA together with tract parameterization analysis. Our results demonstrated fronto-striatal structural connectivity decline (reduced FA) in normal aging with substantial inter-individual differences. The tract parameterization analysis showed that the along tract FA profiles were characterized by piece-wise differential changes along their extension rather than being uniformly affected. To the best of our knowledge, this is the first longitudinal study detecting age-related changes in frontal-subcortical WM connections in normal aging.

## 1. Introduction

It is well-established that normal aging is associated with changes in brain morphometry and white matter (WM) integrity (Salat et al., 2005; Sullivan et al., 2006; Stadlbauer et al., 2008; Davis et al., 2009). However, different areas of the brain seem to undergo changes at different rates and of various extent. Several previous structural magnetic resonance imaging (MRI) studies of aging processes suggest vulnerability of anterior brain regions (Raz et al., 1997, 2007; Allen et al., 2005; Fjell et al., 2010), which is also supported to some degree by diffusion tensor imaging (DTI) findings of age-related decline in frontal WM (Salat et al., 2005; Michielse et al., 2010). These studies have commonly investigated normal cognitive aging with a hypothesis of frontal involvement and have suggested an anterior to posterior gradient of fractional anisotropy (FA) decline (Sullivan et al., 2001; Head et al., 2004; Madden et al., 2004; Sullivan and Pfefferbaum, 2006; Grieve et al., 2007; McLaughlin et al., 2007). These observations may reflect a process of retrogenesis, as anterior brain regions contain a higher fraction of late myelinated fibers compared to posterior regions (Brickman et al., 2012). The hypothesis of WM retrogenesis has also been focused in studies of neuropathological aging (Gao et al., 2011). However, it is still not clarified whether this hypothesis also applies to normal aging (Westlye et al., 2010; Brickman et al., 2012; Kochunov et al., 2012).

Previous cross-sectional studies have shown age-related diffusion differences in distinct WM tracts. In general, the anterior callosal fibers (ACF) (Sullivan et al., 2010) and long association fibers (Stadlbauer et al., 2008; Davis et al., 2009; Michielse et al., 2010; Voineskos et al., 2012) show a more extensive WM integrity decline (i.e., reduction in FA values) compared to projection fibers, although findings are inconsistent (Stadlbauer et al., 2008; Voineskos et al., 2012; Bennett and Madden, 2013). Within the prefrontal cortex (PFC), cross-sectional studies have reported regional differences, revealing more accentuated decline in ventro-medial regions (Raz et al., 2005) and callosal orbitofrontal WM areas (Malykhin et al., 2011) compared to other prefrontal WM areas. Longitudinal age-related changes in the cortico-cortical fibers have previously been revealed over relatively short observation intervals (Barrick et al., 2010; Charlton et al., 2010; Teipel et al., 2010). For subcortico-cortical fibers, however, such longitudinal cohort studies in healthy aging are sparse.

A recent study by Ystad et al. (2011), combining resting state fMRI and DTI, demonstrated that subcortico-cortical fiber tracts connecting functional resting state networks (RSNs) in subcortical nuclei and frontal cortical areas, respectively, could be extracted in the same sample of healthy middle-aged and older adults as the one included in the current study. The structural connectivity of these subcortico-cortical connections were shown to correlate with cognitive measures of executive function in Ystad et al. (2011), thus confirming results from previous studies linking cognitive function to fronto-striatal circuits (Miller and Cohen, 2001; Casey et al., 2002, 2007; Pugh and Lipsitz, 2002; Koechlin et al., 2003; Valente et al., 2005; Liston et al., 2006; Bennett and Madden, 2013). Interestingly, changes in these fronto-striatal networks seem to be one of the characteristics during normal aging (Hedden and Gabrieli, 2004; Fjell et al., 2010; Jagust, 2013). However, these networks are also affected by neurodegenerative diseases, at least in the more advanced stages (Zhang et al., 2009; Wilson et al., 2012; Jagust, 2013). Thus, follow-up studies, describing the course and amount of age-related changes in a given person, provide a valuable complement to cross-sectional studies in distinguishing healthy individuals from those with impending disease. This makes further longitudinal studies of the fronto-subcortical connectivity in healthy older adults warranted.

**Figure 1:**
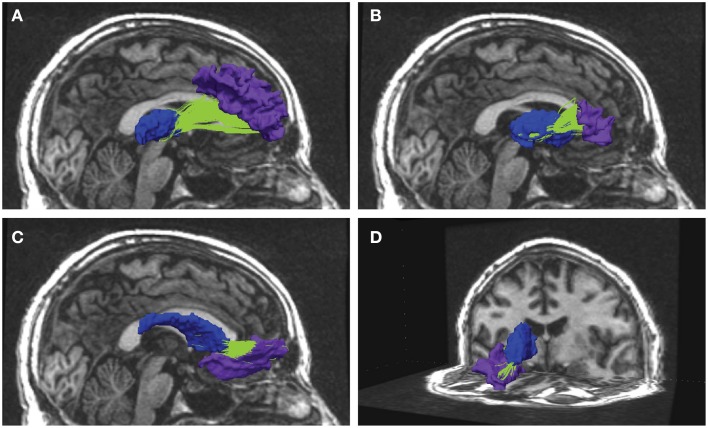
**Examples from reconstructed frontal-subcortical fiber bundles in right hemisphere**. The images show four different frontal-subcortical fiber bundles reconstructed in one of the participants from the multimodal MRI recordings at Wave-2. The selected WM tracts (in green) are co-registered with and superimposed on the 3D T1-weighted images, where the source and target regions obtained from FreeSurfer segmentation are color-coded. Blue segment represents subcortical seed region and purple segment represents frontal WM target region. Fiber bundle connections in the right hemisphere are running between: **(A)** thalamus and rostral middle frontal region; **(B)** putamen and the pars triangularis; **(C)** caudate nucleus and lateral orbitofrontal region; **(D)** caudate nucleus and medial orbitofrontal region.

In the present longitudinal study, anatomical segmentation from 3D T1-weighted recordings were combined with diffusion weighted (DW) MRI-based tractography to extract frontal-subcortical fiber bundles (illustrated in Figure 1). More specifically, fiber connections that are running between subcortical seed regions of the striatum, thalamus, and nucleus accumbens, and a wide range of segmented WM subregions in the frontal lobes. The aim was to investigate (i) the anatomical distribution of FA changes, and (ii) the rate and degree of FA change (i.e., tract overall mean FA, and along-tract FA) in the frontal fiber bundles across a time period of about 7 years. The major fibers of ACF and the corticospinal tract (CST) were identified to serve as control tracts for the smaller and less studied frontal-subcortical fiber bundles. Based on previous reports we expected an age-related FA reduction in the ACF (Sullivan et al., 2006, 2010) and in frontal-subcortical fibers (Fjell et al., 2010; Jagust, 2013), whereas the CST was assumed to show a more stabile FA across time (Stadlbauer et al., 2008; Perry et al., 2009; Sullivan et al., 2010; Losnegård et al., 2013).

The main contributions of the present study are to provide longitudinal data on the integrity of subcortico-cortical fibers together with local information about water diffusion measures along normalized segments of the individual fiber tracts using tract parameterization in a cohort of healthy middle-aged and older participants. Repeated MRI investigations in the same individual across a time span of several years, together with tractography and along-tract analysis, will thus yield valuable information about consistency and trends in WM integrity (FA) across tracts, subjects, and age, where results are likely influenced by both methodological variations and biological processes. The study is thus an important supplement to cross-sectional overall mean FA analyses, and provides more comprehensive spatio-temporal information about the aging process in frontal WM (Davis et al., 2009; Colby et al., 2012; Hodneland et al., 2012) than could be obtained from cross-sectional studies of comparable size, alone.

## 2. Materials and methods

### 2.1. Participants

The present study included 76 healthy older Norwegian participants (52 females) who took part in a longitudinal study on cognitive aging. Demographic data are summarized in Table 1. The participants were all recruited through local advertisements, they provided blood-samples for genetic analysis and were subject to neuropsychological and MRI examinations. None of them reported present or previous neurological or psychiatric disorders, a history of substance abuse or other significant medical conditions. An experienced neuropsychologist (AJL) evaluated the results from from the neuropsychological examination, including a set of standardized tests of sensori-motor function, attention/executive function, memory, language function and visual cognition, and confirmed that none of the participants were demented or showed symptoms indicating mild cognitive impairment (MCI) (Petersen, 2004). The MRI examination did not show structural abnormalities such as tumor, stroke, extensive WM hyperintensity or other anatomical variations that made morphometric analysis inaccurate. All participants took part in a second examination (study wave, or “wave” for short) 3–4 years later, mean 3.6 years. Neuropsychological and MRI data were obtained according to a similar protocol as in the first wave, extended by including the Mini Mental State Examination (MMSE) (Folstein et al., 1975). Their scores on MMSE were all greater than 26 (range 26–30), indicating no obvious signs of dementia.

**Table 1:** **Demographic characteristics of the cohort**.

	**Wave-1**	**Wave-2**	**Wave-3**
Participants (N)	76	76	24
Age [years] (mean ± SD)	59 ± 7	62 ± 7	63 ± 6
Age range [years]	46–78	49–80	52–78
Sex (F:M)	52:24	52:24	9:15
MMSE score (mean ± SD)	–	28.9 ± 0.99	28.9 ± 1.50
Education [years] (mean ± SD)	14 ± 3	14 ± 3	14 ± 3
Education range [years]	9–20	9–20	10–19

*MMSE = Mini Mental State Examination*.

Twenty-four of the subjects (nine females) did also participate in a third wave, 3–4 years (mean 3.6) after the second one. Their scores on the MMSE were equal to or greater than 25 (range 25–30). The neuropsychological and MRI examination protocols were similar to the ones used in the two earlier waves.

Three participants were excluded from the longitudinal analyses: two because they were diagnosed with Parkinson's Disease, and one due to a low MMSE score (23) in the third wave. All participants signed an informed consent form, and the Regional Committees for Medical and Health Research Ethics of southern (wave 1) and western (wave 2 and 3) Norway approved the study.

### 2.2. Data acquisition

Multimodal MRI examinations, both at baseline and at the follow-ups, were performed on the same 1.5 T GE Signa Echospeed scanner (MR laboratory, Haraldsplass Deaconess Hospital, Bergen) using a standard 8-channel head coil. No major scanner upgrades were done between wave 1 and wave 3. The 3D T1-weighted and the DWI data were acquired during the same imaging session, without repositioning the subject, allowing the positional information in the DICOM file headers to be used in subsequent multimodal image registration. At each wave, two consecutive T1-weighted 3D volumes were recorded using a fast spoiled gradient echo (FSPGR) sequence. DWI were collected using a spin echo echo planar (SE-EPI) sequence including 25 directions with *b* = 1000 s/mm^2^; 5 images with *b* = 0; for all three waves. Details about the MRI pulse sequences are given in Table 2.

**Table 2:** **The multimodal MRI protocols used in each of the three waves (2005, 2008, and 2011)**.

**MRI series**	**Wave-1**	**Wave-2**	**Wave-3**
3D T1-WEIGHTED			
TR/TE/TI/FA	9.45/2.41/450/7	9.1/1.77/450/7	same as wave 2
Ax|VOX	124|0.94×0.94×1.4	same as wave 1	same as wave 1
DWI			
TR/TE/FA	7900/97.5/90	7900/106.8/90	7900/107.9/90
Ax|VOX|IM	26|0.94×0.94×4.0|780	25|0.94 × 0.94 × 4.0|750	same as wave 2

*TR/TE/TI/FA = repetition time [ms]/echo time [ms]/inversion time [ms]/flip angle [deg]. Ax|VOX|IM = number of axial slices | voxel size [mm^3^] | number of images in total*.

### 2.3. Anatomical segmentation

The software package FreeSurfer version 4.3.1 (http://surfer.nmr.mgh.harvard.edu) was used for structural image analysis at all three waves. The two 3D T1 weighted volumes recorded in succession were first coregistered and averaged to improve signal to noise ratio (SNR). The following semi-automated FreeSurfer processing pipeline included Talairach registration (Talairach and Tournoux, 1988), skull stripping (Ségonne et al., 2004), intensity normalization, tissue segmentation, and cortical reconstruction. Each analyzing step, including automatic anatomical labeling, was carefully inspected, and manual edits were preformed if needed. The FreeSurfer parcellated and labeled WM regions and subcortical nuclei were used as a basis for defining regions of interests (ROIs) in the subsequent DWI-based fiber tracking/WM analyses.

### 2.4. DW image processing

#### 2.4.1. DTI reconstruction and fiber tracking

An in-house MATLAB-program was implemented to automate the workflow of tensor reconstruction, FA estimation, and fiber tracking from the DWI recordings and to perform multimodal MRI registration (for more details, see Hodneland et al., 2012). The first step in DTI data processing incorporated eddy current correction of the DWI images, using image registration routines from the FSL package (http://fsl.fmrib.ox.ac.uk/fsl/fslwiki/). The Diffusion Toolkit software package (Wang et al., 2007, www.trackvis.org) was then used for tensor reconstruction from the DWI images and voxel-wise calculation of the eigenvalues and eigenvectors of the tensor (Basser et al., 1994). The FA maps were constructed and the fiber assignment by continuous tracking (FACT) algorithm was employed for tractography (Mori and van Zijl, 2002). For each subject, the calculated FA images and the fiber tracts were non-linearly registered to the subjects high-resolution 3D anatomical space (Hodneland et al., 2012). A non-linear registration was required in order to reduce geometric distortions arising between the DWI EPI recordings and the 3D T1-weighted recordings. Our non-linear registration utilizes normalized gradients enforcing collinear gradients between the input and target image, ensuring that the structural boundaries occur at the same locations in the input and in target image. This multimodal MRI registration procedure allowed for extracting tracts of interest (TOI) by intersecting the spatially aligned tract volume with selected ROIs defined by FreeSurfer.

**Figure 2:**
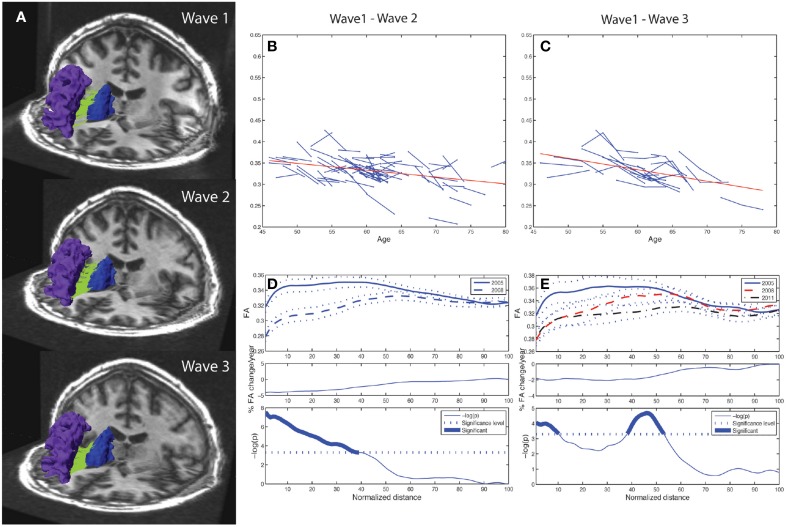
**Tract mean FA and along-tract FA profiles of right caudate to rostral middle frontal fiber bundle**. Depiction of the reproducibility of frontal-subcortical fiber bundle reconstructions across three different time points (i.e., right caudate to rostral middle frontal fiber bundle for one of the participants being examined in 2005, 2008, and 2011). **(B)** Line graph (spaghetti) plot for the cohort (*N* = 76), between Wave-1 and Wave-2, of tract mean FA of left caudate to rostral middle frontal fiber bundle. A least-squares fitted line (red) for the pooled Wave-1 and Wave-2 observations is overlayed, and shows age-related reduction in tract mean FA. **(C)** Line graph plot for the subset (*N* = 24) of participants being scanned three times, i.e., Wave-1, Wave-2, and Wave-3, of tract mean FA of the fiber bundle illustrated in **(A)**. A least-squares fitted line (red) for the pooled Wave-1, Wave-2, and Wave-3 observations is overlayed, and shows further age-related reduction in tract mean FA. **(D)** Upper panel: Along-tract FA profiles of fiber bundle illustrated in **(A)**, i.e., segment-wise mean FA (continuous line in Wave-1, broken line in Wave-2) and ± 1 SE (dotted lines above and below the mean profile), across the cohort. Note that the fiber bundle is length-normalized into 100 equidistant points along the tract. Middle panel: Average percentage change per year of FA at each segment along the fiber bundle, from Wave-1 to Wave-2, across cohort. Lower panel: Segments for which there is a significant (Bonferroni corrected *p* < 0.001, paired *t*-test) change in FA along the fiber, highlighted with a thicker line. The *p*-value is scaled as −log10 (p). **(E)** Upper panel: Along-tract FA profiles of fiber bundle illustrated in **(A)** for the subset (*N* = 24) having three MRI examinations, i.e., segment-wise mean FA (continuous line in Wave-1, broken line in Wave-2, mixed broken & dotted line in Wave-3) and ± 1 SE (dotted lines), across the cohort. Middle panel: Average percentage change per year of FA at each segment along the fiber bundle, from Wave-1 to Wave-3, across subsample. Lower panel: Segments for which there is a significant (Bonferroni corrected *p* < 0.001, repeated measures ANOVA) change in FA along the fiber, highlighted with a thicker line. The *p*-value is scaled as −log10(*p*).

The algorithm used tracts from the entire volume and selected those specific tracts fulfilling the criteria of running between or crossing one or more of the desired ROIs (Huang et al., 2004; Hodneland et al., 2012). ROI for extracting the fiber tracts were based on anatomical guidelines and published reports including a diffusion tractography atlas (Wakana et al., 2004). For investigating ACF and the fiber bundles connecting subcortical and frontal WM areas, a source and target approach was used. The subcortical structures putamen, caudate, pallidum, thalamus and accumbens were considered as separate seed areas. Further, the following 22 automatically labeled WM areas (11 in each hemisphere), covering the frontal lobe, were defined as targets for the tracking (Desikan et al., 2006); superior frontal, anterior cingulate (caudal anterior cingulate and rostral anterior cingulate), orbitofrontal (lateral orbitofrontal and medial orbitofrontal), frontal pole, inferior frontal (pars orbitalis, pars triangularis, and pars opercularis), and the middle frontal (caudal middle and rostral middle). The areas of dorsolateral (including rostral middle frontal and pars triangularis), caudate and putamen were regarded as structures of the fronto-striatal circuits. Trackvis (www.trackvis.org) was used for visualizing the tracts. Figure 2A illustrates how consistent the automated procedure could identify a given fiber connection (the one between the caudate nucleus and the rostral middle frontal WM area of the right hemisphere) in a given individual across three MRI examinations, spanning 7 years observation time. In each wave, a threshold of minimum 20 continuous fibers was set for the fiber bundles to be recognized and included as a proper tract. As a requirement for inclusion in further analyses the fiber connection had to be identified in at least 90% of the subjects. Fiber connections fulfilling these threshold criteria are described in Table 3 indicated by bold values.

**Table 3:** **Number of subjects (maximum is 76) for which structural connections could be identified, according to the criteria described in the text, between seed ROIs representing subcortical nuclei (left column) and 11 target ROIs in frontal white matter areas (top row): superior frontal (SF), anterior cingulate [caudal anterior cingulate (CAC), rostral anterior cingulate (RAC)], orbitofrontal [lateral orbitofrontal (LOF), medial orbitofrontal (MOF)], frontal pole (FP), inferior frontal [pars orbitalis (PO), pars trinagularis (PT), pars opercularis (POC)], and the middle frontal [caudal middle (CMF) and rostral middle (RMF)]**.

**FreeSurfer regions**	**SF**	**CAC**	**RAC**	**MOF**	**LOF**	**FP**	**PO**	**PT**	**POC**	**RMF**	**CMF**
Left thalamus	**69**	38	11	44	43	4	43	6	4	**71**	38
Right thalamus	66	45	7	18	44	4	5	24	7	73^*^	48
Left caudate	**71**	34	54	72^*^	72^*^	29	11	38	3	72^*^	14
Right caudate	53	25	16	71^*^	72^*^	38	2	8	NA	74^*^	5
Left putamen	**69**	25	18	**74**	**76**	17	62	**73**	48	**74**	61
Right putamen	50	15	5	67	75^*^	37	61	75^*^	46	75^*^	42
Left pallidum	35	1	NA	18	40	NA	7	32	NA	45	7
Right pallidum	40	13	NA	9	50	NA	4	32	7	61	28
Left accumbens	NA	NA	24	**70**	61	2	NA	NA	NA	NA	NA
Right accumbens	NA	16	NA	64	64	2	NA	NA	NA	2	NA

*NA, not available; bold values indicate connections fulfilling the threshold criteria*.

* *Entries where the connection has a significant mean FA change between Wave-1 and Wave-2 (as shown in **Table 4**)*.

#### 2.4.2. White matter integrity analysis including tract parameterization

For a given participant and WM tract, the FA values were initially taken as the grand average of the FA map region that intersected the specific fiber tract. This is the most frequently used method to assess tract-specific water diffusion properties. To obtain more local information, we applied a method of tract parameterization, which calculates FA values in discrete segments along the tract (Davis et al., 2009; Colby et al., 2012; Hodneland et al., 2012). Two different approaches were used in tract-parameterzation analysis. For the subcortical tracts, traversing between two regions, we used length-normalized parameterization. These fiber tracts were divided into 100 equidistant data points starting in the seed region (data point number 1) progressing along their path to the target area (data point number 100). The ACF were parameterized into 2 × 100 equidistant segments, 100 segments in each hemisphere. This approach of tract parameterization between two ROIs was in accordance with previous studies (Lin et al., 2006; Oh et al., 2007, 2009). For the CST, we used the WM region of the precentral gyrus as a defining ROI. For further details see (Hodneland et al., 2012).

### 2.5. Statistical analysis

A paired-sample *t*-test was used to investigate changes between wave 1 and wave 2. In this analysis the possible effect of age variability at inclusion is not taken into account. To better account for the age effect in the cohort, a line-graph plot was used to visualize the change in mean FA between two time-points, for a given participant and a given tract (cf. Figure 2). To explore whether age at inclusion had an impact on the yearly change in FA, we computed the Pearson correlation between age at wave 1 and yearly change in FA from wave 1 to wave 2. We found no significant correlation, indicating that the yearly change in FA was relatively constant across the age distribution in our sample. If a subject had an FA increase or decrease in a given tract larger that 2.5 standard deviations with respect to the variation in the whole sample, such tract measures were considered as outliers and were excluded in the final analyses (cf. Tables 4, **6**). The mean percentage change was first calculated for each subject and then averaged for the total sample; the latter one is presented in the Tables 4–**7**. In the tract parameterization analysis we segment-wise calculated mean FA ± standard error (SE) and percentage rate of change in FA per year, and performed paired *t*-test analysis (cf. Figures 2D, **4A,B, 5A,B** and Supplementary Figures 1, 2).

**Table 4:** **Mean and standard deviation of tract mean FA in the 19 extracted fiber connections (A)–(S) (column 1) that were detected in at least 90% of the participants: at baseline (column 2) and at follow-up 3 years later (column 3)**.

**Connection**	**Wave-1**	**Wave-2**	***P*-value**	***N***	**% ΔFA/year**	***M***
(A) LH Thal ↔ SF	0.387 ± 0.035	0.379 ± 0.030	0.376	69	0.17	1
(B) LH Ca ↔ SF	0.346 ± 0.045	0.327 ± 0.038	0.024	71	−0.82	2
(C) LH Pu ↔ SF	0.377 ± 0.034	0.363 ± 0.032	0.917	69	−0.02	0
(D) LH Ca ↔ MOF	0.315 ± 0.025	0309 ± 0.025	**<0.005**	72	−0.92	4
(E) RH Ca ↔ MOF	0.322 ± 0.034	0.319 ± 0.031	**<0.001**	71	−1.24	3
(F) LH Pu ↔ MOF	0.327 ± 0.027	0.321 ± 0.030	0.329	74	−0.20	2
(G) LH Acc ↔ MOF	0.260 ± 0.040	0.259 ± 0.033	0.089	70	−0.66	0
(H) LH Ca ↔ LOF	0.338 ± 0.026	0.325 ± 0.025	**<0.001**	72	−1.70	3
(I) RH Ca ↔ LOF	0.339 ± 0.024	0.326 ± 0.027	**<0.001**	72	−2.09	4
(J) LH Pu ↔ LOF	0.356 ± 0.024	0.357 ± 0.024	0.767	76	0.02	0
(K) RH Pu ↔ LOF	0.362 ± 0.030	0.359 ± 0.025	**<0.001**	75	−1.03	1
(L) LH Pu ↔ PT	0.369 ± 0.026	0.371 ± 0.028	0.526	73	0.20	3
(M) RH Pu ↔ PT	0.364 ± 0.028	0.357 ± 0.027	**<0.001**	75	−1.03	0
(N) LH Thal ↔ RMF	0.349 ± 0.029	0.344 ± 0.030	0.048	71	−0.64	0
(O) RH Thal ↔ RMF	0.373 ± 0.028	0.364 ± 0.030	**<0.001**	73	−1.67	2
(P) LH Ca ↔ RMF	0.329 ± 0.025	0.321 ± 0.025	**<0.001**	72	−1.64	4
(Q) RH Ca ↔ RMF	0.338 ± 0.037	0.332 ± 0.028	**<0.001**	74	−1.56	1
(R) LH Pu ↔ RMF	0.352 ± 0.023	0.353 ± 0.026	0.629	74	−0.04	2
(S) RH Pu ↔ RMF	0.361 ± 0.024	0.353 ± 0.026	**<0.001**	75	−0.91	1

*The percentage annual change of mean FA (calculated first for each subject and then averaged across the total sample) is given in column 6. N (≤ 76; column 5) denotes the total number of subjects for which the specific fiber bundle could be identified according to the given criteria. M (column 7) denotes the number of participants discarded by the exclusion criteria of having tract mean FA larger than 2.5 SD with respect to the FA variation in the whole sample. P-value (column 4) is calculated from paired-samples t-test assuming null hypothesis of no change in tract mean FA between Wave-1 and Wave-2. Significant change is marked by bold values. LH, left hemisphere; RH, right hemisphere; Acc, accumbens area; Ca, caudate; Pu, putamen; Thal, thalamus; SF, superior frontal; MOF, medial orbitofrontal; LOF, lateral orbitofrontal; PT, pars triangularis; RMF, rostral middle frontal*.

To analyze the three wave longitudinal changes in the subsample, repeated-measures ANOVA was carried out both for mean FA and for segment-wise FA. As for the two-wave analysis, the overall change in mean FA between the three time points was fitted with a linear regression line of the mean change for all the subjects. Further, the average annual percentage FA change was calculated over these two intervals.

MATLAB was used in the statistical analyses. To correct for type 1 error associated with multiple comparisons we used Bonferroni method. Corrected alpha level of 0.0023 was set for the mean FA analyses after correcting for 22 multiple comparisons (the number of frontal-subcortical fibers, ACF and left & right CST). For the tract parameterization analyses, corrected alpha level was set to 0.0005 after correcting for 100 multiple comparisons (the number of segments in one tract).

## 3. Results

### 3.1. Identification, reconstruction, and assessment of frontal-subcortical fibers

Our combined segmentation and fiber reconstruction methods provided identification, reconstruction, and information about the FA properties of several frontal-subcortical fiber connections. Examples of the reconstructed frontal-subcortical fibers are visualized in Figure 1. The number of subjects for which we could identify and characterize connections between subcortical nuclei and target ROIs in frontal WM is presented in Table 3. In this framework, the maximum number of extracted fiber bundles in a given subject was 110. However, only 19 (marked by bold values) of the investigated connectivity combinations were large enough (minimum of 20 continuous fibers) to be detected in at least 90% of the subjects, and by this fulfilling the criteria of being included in further analysis.

Striatum, including caudate and putamen, exhibited the highest number of frontal connections. Connectivity between the caudate nucleus extended especially to the superior frontal, the lateral, and medial orbitofrontal, and the rostral middle frontal WM areas. Putamen had projections to the same frontal areas as the caudate nucleus, with the addition of the pars triangularis. Thalamus had fewer connections to orbitofrontal areas compared to the striatum. With the thalamus as seed region, superior frontal, and rostral middle frontal areas seemed to be those target areas exhibiting the highest numbers of connections. Fiber bundles arising from nucleus accumbens as the seed region had distinct connections to the orbitofrontal areas (mainly found in between 80 and 90% of the subjects), leaving other frontal areas without detectable connections. Still, only the connection to the left medial orbitofrontal area fulfilled the threshold criterion. Sparse connections were seen between pallidum and the frontal WM areas, not fulfilling the threshold criteria.

### 3.2. Two wave FA changes in frontal-subcortical fiber bundles

Ten of the 19 fiber connections being included according to the selection criteria revealed significant mean FA decrease from Wave-1 to Wave-2 (Table 4; connections D, E, H, I, K, M, O, P, Q, and S). The mean percentage annual decrease in mean FA of these connections was 1.38%, ranging from a minimum decrease of 0.91% (right putamen to rostral middle frontal WM area) to a maximum decrease of 2.09% (right caudate to lateral orbitofrontal WM area). Several connections with seed regions in the thalamus, the caudate, and the putamen projecting to target areas in frontal WM regions (details in Table 4), exhibited significant reduction in mean FA. Superior frontal region was the only target area that did not have any subcortical connections showing significant decline (Table 4; connections A, B, and C).

While the 10 frontal-subcortical connections revealed significant decline in tract mean FA with age (illustrated in Figures 2B,C), the along-tract FA profiles were not uniformly affected, but showed piece-wise differential changes along their extension (cf. Figures 2D,E). The least-squares fitted line in Figure 2B reveals an overall negative trend in mean FA from Wave-1 to Wave-2, and Figure 2C shows a similar negative trend in mean FA for the subset of 24 participants having three MRI examinations. In Figures 2D,E the along-tract segments showing significant decrease in FA (averaged across the cohort) between study waves are highlighted with a thicker line in the lower panels. The middle panels show the annual percentage rate of change along the tract. Tract-wise FA profile changes for the remaining nine subcortico-frontal fiber connections are presented in the Supplementary Figure 1. In brief, these connections showed large variations in segment-wise FA change along the fiber bundle, where a typical pattern was largest FA change in either the initial part, the terminal part, or in both ends of the fiber bundle.

### 3.3. Three wave FA changes in frontal-subcortical fiber bundles

Generally, the three-wave analyses, applicable to a subset of 24 participants, showed a more moderate percentage annual reduction of tract mean FA compared to the two-time point analysis of the whole cohort. Across the 19 different subcortico-cortical connections and three study waves, we found a minimum decrease of 0.07% (left putamen to pars triangularis) and a maximum decrease of 1.71% (left and right caudate to lateral orbitofrontal regions), with a mean of 0.82% decrease in tract mean FA each year (Table 5).

**Table 5:** **Mean and standard deviation of tract mean FA in the 19 extracted fiber connections (A)–(S) (column 1) that were detected in the subsample of 24 participants having three MRI examinations: at baseline (column 2), at first follow-up (column 3) and at second follow-up (column 4)**.

**Connection**	**Wave-1**	**Wave-2**	**Wave-3**	***P*-value**	***N***	**% ΔFA/year**
(A) LH Thal ↔ SF	0.386 ± 0.033	0.376 ± 0.022	0.384 ± 0.032	0.922	21	−0.19
(B) LH Ca ↔ SF	0.350 ± 0.042	0.325 ± 0.036	0.331 ± 0.029	0.150	19	−0.43
(C) LH Pu ↔ SF	0.384 ± 0.031	0.368 ± 0.032	0.365 ± 0.024	0.229	20	−0.25
(D) LH Ca ↔ MOF	0.320 ± 0.021	0.309 ± 0.023	0.308 ± 0.029	**<0.001**	24	−1.45
(E) RH Ca ↔ MOF	0.326 ± 0.030	0.323 ± 0.026	0.315 ± 0.029	**<0.001**	23	−1.48
(F) LH Pu ↔ MOF	0.334 ± 0.022	0.323 ± 0.029	0.322 ± 0.036	0.006	20	−1.11
(G) LH Ac ↔ MOF	0.262 ± 0.041	0.261 ± 0.032	0.244 ± 0.035	0.214	19	−0.28
(H) LH Ca ↔ LOF	0.343 ± 0.023	0.324 ± 0.026	0.325 ± 0.027	**<0.001**	24	−1.71
(I) RH Ca ↔ LOF	0.339 ± 0.021	0.332 ± 0.025	0.331 ± 0.024	**<0.001**	24	−1.71
(J) LH Pu ↔ LOF	0.360 ± 0.022	0.361 ± 0.225	0.363 ± 0.023	0.098	24	−0.46
(K) RH Pu ↔ LOF	0.366 ± 0.024	0.364 ± 0.020	0.362 ± 0.018	0.019	24	−0.50
(L) LH Pu ↔ PT	0.371 ± 0.023	0.378 ± 0.030	0.380 ± 0.025	0.793	24	−0.07
(M) RH Pu ↔ PT	0.369 ± 0.021	0.363 ± 0.021	0.366 ± 0.021	**<0.005**	24	−0.52
(N) LH Thal ↔ RMF	0.359 ± 0.025	0.348 ± 0.025	0.349 ± 0.026	0.052	19	−0.45
(O) RH Thal ↔ RMF	0.375 ± 0.019	0.367 ± 0.029	0.369 ± 0.027	**<0.001**	22	−1.02
(P) LH Ca ↔ RMF	0.334 ± 0.020	0.321 ± 0.019	0.324 ± 0.018	**<0.001**	24	−1.29
(Q) RH Ca ↔ RMF	0.338 ± 0.024	0.333 ± 0.030	0.335 ± 0.022	**<0.001**	24	−1.42
(R) LH Pu ↔ RMF	0.355 ± 0.019	0.356 ± 0.023	0.355 ± 0.024	0.077	24	−0.45
(S) RH Pu ↔ RMF	0.363 ± 0.023	0.358 ± 0.020	0.356 ± 0.019	**<0.001**	24	−0.75

*N (≤ *24*; column 6) denotes the total number of subjects for which the specific fiber bundle could be identified according to the given criteria. See Table 4 for further descriptions*.

As illustrated in Figure 2E for the right caudate to rostral middle frontal fiber bundle, the tract parameterization results revealed a trend of FA reduction from the first to the second wave and further reduction was shown in the third wave. The age-related pattern of along-tract FA changes appeared to be similar to that observed in the two-wave analysis. However, the changes were more moderate (data for the remaining nine fiber bundles are not shown).

### 3.4. Anterior callosal fibers and the corticospinal tract

**Figure 3:**
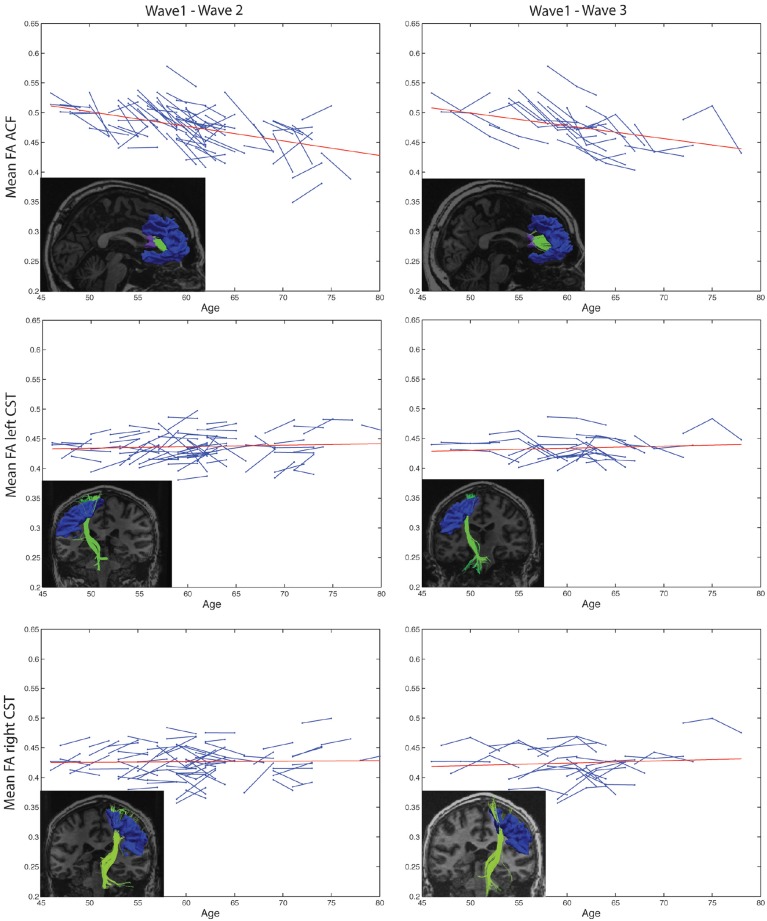
**Subject-specific longitudinal tract mean FA in anterior callosal fiber (ACF) bundle, and left and right corticospinal tract (CST) during aging**. Left column: Tract mean FA for each participant at Wave-1 and Wave-2. Right column: Tract mean FA for the subset of participants having three MRI examinations, i.e., at Wave-1, Wave-2, and Wave-3. For each of the tracts, a linear regression line (red) of the pooled tract mean FA across all subjects and study waves are superposed on the spaghetti plots. The inserted images illustrate the particular tract, ACF, left CST, or right CST, reconstructed at Wave-1 (left column) and at Wave-3 (right column) in one of the participants.

Highly significant age related longitudinal decrease in mean FA was found bilaterally in the ACF. In the full cohort, the annual mean FA reduction in this fiber bundle was −1.50% across Wave-1 to Wave-2 (Table 6), and 1.34% across Wave-1 to Wave-3 in the subsample of 24 participants (Table 7). A minor (not statistically significant) increase in tract mean FA was found in the CST between Wave-1 and Wave-2, and with inclusion of the subsample having three MRI examinations we found a slight annual FA decrease of −0.23% in left hemisphere CST (Table 7). In more detail, Figure 3 shows the individual tract mean FA changes during aging for the ACF, the left CST, and the right CST, respectively. The left column in Figure 3 provides tract-wise FA changes for each participant having two MRI examinations, and the right column FA changes for the subset of participants having three MRI examinations, i.e., at Wave-1, Wave-2, and Wave-3. Three major findings, illustrated in Figure 3 can be summarized as follows: (i) there are substantial intra and inter-individual variations in the tract-wise mean FA with time (spaghetti plots); for the cohort in total (regression lines) there are (ii) an almost stable mean FA in the left and right CST during the observed age-span; this in contrast to (iii) a substantial decrease in mean FA of the ACF bundle with age.

**Table 6:** **Mean and standard deviation of tract mean FA in the anterior callosal fiber (ACF) bundle, and in left and right hemispheres (LH, RH) of the corticospinal tract (CST) at baseline (Wave-1) and at follow-up 3 years lager (Wave-2)**.

**Tract**	**Wave-1**	**Wave-2**	***P*-value**	***N***	**% ΔFA/year**	***M***
ACF	0.437 ± 0.033	0.420 ± 0.028	**<0.001**	74	−1.50	1
LH CST	0.434 ± 0.025	0.439 ± 0.023	0.014	76	0.49	0
RH CST	0.424 ± 0.027	0.427 ± 0.028	0.058	74	0.36	2

*The percentage annual change of tract mean FA (calculated first for each subject and than averaged across the total sample) is given in column 6. N (≤ *24*; column 6) denotes the total number of subjects for which the specific fiber bundle could be identified according to the given criteria. M (column 7) denotes the number of participants discarded by the exclusion criteria of having tract mean FA larger than 2.5 SD with respect to the FA variation in the whole sample. P-value (column 4) is calculated from paired-samples t-test assuming null hypothesis of no change in tract mean FA between Wave-1 and Wave-2. Significant change is marked by bold values*.

**Table 7:** **Mean and standard deviation of tract mean FA in the in the anterior callosal fiber (ACF) bundle, and in left and right hemispheres (LH, RH) of the corticospinal tract (CST) that were detected in the subsample of 24 participants having three MRI examinations: at baseline (column 2), at first follow-up (column 3), and at second follow-up (column 4)**.

**Tract**	**Wave-1**	**Wave-2**	**Wave-3**	***P*-value**	***N***	**% ΔFA/year**
ACF	0.446 ± 0.027	0.423 ± 0.024	0.419 ± 0.029	**<0.001**	24	−1.34
LH CST	0.434 ± 0.022	0.439 ± 0.018	0.432 ± 0.017	**<0.001**	24	−0.23
RH CST	0.421 ± 0.031	0.430 ± 0.030	0.427 ± 0.028	0.016	24	0.10

*N (≤ *24*; column 6) denotes the total number of subjects for which the specific fiber bundle could be identified according to the given criteria. P-value (column 5) is calculated from repeated measures ANOVA across Wave-1, Wave-2, and Wave-3. Significant change is marked by bold values. Column 7 provide percentage annual change of tract mean FA (calculated first for each subject and than averaged across the total sample)*.

**Figure 4:**
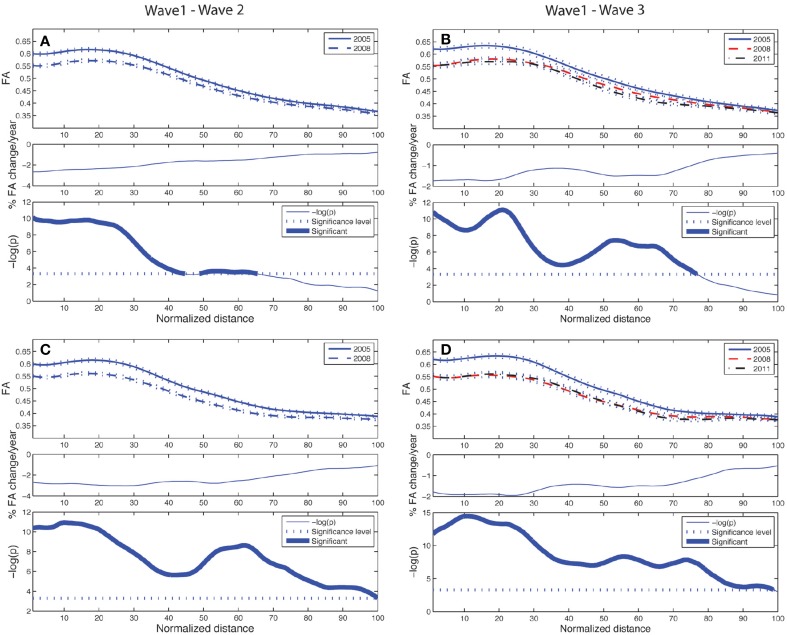
**Tract parameterization of the ACF for the two-wave (left column) and the three-wave (right column) analyses**. The individual trajectories of the ACF is normalized into 100 equidistant segments, starting in the genu of corpus callosum (segment 1) traversing to the left and right prefrontal WM areas, respectively (segment 100). **(A)** Upper panel: Mean FA ± SE (dotted lines) profile of left hemispheric branch of ACF at Wave-1 (continuous line) and Wave-2 (broken line). Middle panel: Segment-wise percentage change in mean FA per year in the cohort from Wave-1 to Wave-2. Lower panel: Segment-wise significance in mean FA difference between Wave-1 and Wave-2 in the cohort. The *p*-value is scaled as −log(p). Fat parts of the line denote ACF segments with statistically significant (Bonferroni corrected *p* < 0.001, paired *t*-test) FA change. **(B)** Upper panel: Mean FA ± SE (dotted lines) profile of left hemispheric branch of ACF in participants having three MRI examinations, Wave-1 (continuous line), and Wave-2 (broken line), Wave-3 (broken & dotted line). Middle panel: Segment-wise percentage change in mean FA per year in the subsample from Wave-1 to Wave-3. Lower panel: Segment-wise significance in FA difference between Wave-1 and Wave-3. The *p*-value is scaled as −log(p). Fat parts of the line denote segments with statistically significant (Bonferroni corrected *p* < 0.001, repeated measures ANOVA) FA change. **(C)** Same as in **(A)** for right hemispheric branch of the ACF. **(D)** Same as in **(B)** for right hemispheric branch of the ACF.

**Figure 5:**
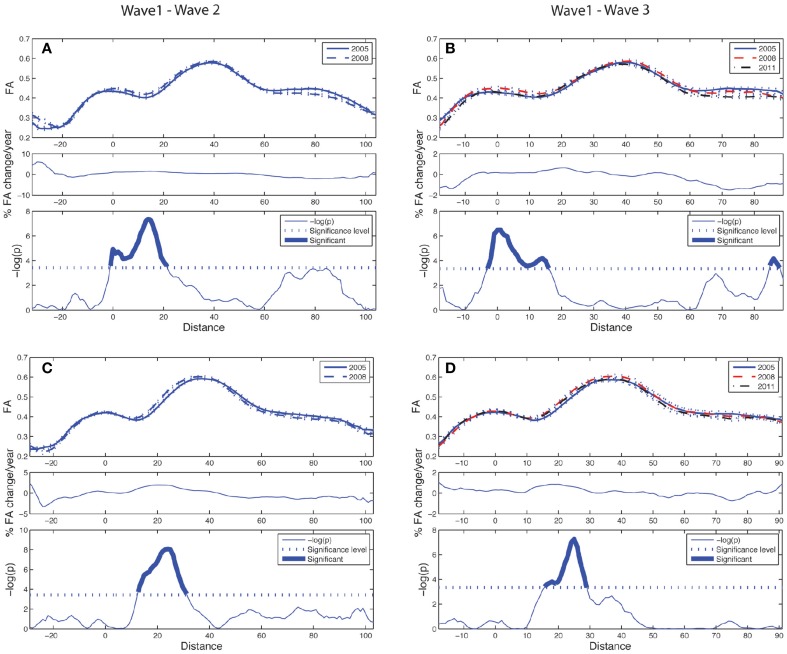
**Tract parameterization of the CST for the two-wave (left column) and the three-wave (right column) analyses**. The individual trajectories of the CST, initialized from only one ROI, is normalized into 100 equidistant segments where 0 on the normalized distance axis is located along the CST in the middle part of the precentral WM region, and segment 100 is at a cut at the lower level of the brainstem (cf. image inserts in lower parts of left and right column in Figure 3). **(A)** Upper panel: Mean FA ± SE (dotted lines) profile of left hemispheric CST at Wave-1 (continuous line) and Wave-2 (broken line). Middle panel: Segment-wise percentage change in mean FA per year in the cohort from Wave-1 to Wave-2. Lower panel: Segment-wise significance in mean FA difference between Wave-1 and Wave-2 in the cohort. **(B)** Upper panel: Mean FA ± SE (dotted lines) profile of left hemispheric CST in participants having three MRI examinations, Wave-1 (continuous line), and Wave-2 (broken line), Wave-3 (broken & dotted line). Middle panel: Segment-wise percentage change in mean FA per year in the subsample from Wave-1 to Wave-3. Lower panel: Segment-wise significance in FA difference between Wave-1 and Wave-3. **(C)** Same as in **(A)** for right hemispheric CST. **(D)** Same as in **(B)** for right hemispheric CST. See Figure 4 for further details.

The longitudinal tract parameterization analysis of FA along the ACF bundle is depicted in Figure 4. The most substantial and significant decrease in segment-wise FA with age is found in the the central part of the bundle, while the FA decrease was less significant toward the cortical sheet. This FA reduction in ACF with age was slightly more pronounced in the right hemisphere (Figures 4C,D) compared to the left (Figures 4A,B). The longitudinal tract parameterization analysis of FA along the CST is depicted in Figure 5. In summary, the along-tract changes in FA between the study waves were much smaller, and spatially less consistent, than for the ACF bundle. Only CST segments in the distance range 0–30 close to the precentral WM region (thicker lines in lower panels in Figure 5), showed significant but small aging-related FA decrease.

## 4. Discussion

To the best of our knowledge, this is the first longitudinal investigations of FA time courses obtained for a large set of frontal-subcortical fiber tracts in healthy aging across an observation time of at least 7 years. Using multimodal image registration, image segmentation, and tractography, we were able to identify frontal-subcortical fiber connections between 11 cortical regions and five subcortical structures in each hemisphere, and further address changes in tract-wise mean FA and local segment-wise FA. A major finding was age-related reduction in tract-wise FA, confirming previous reports from cross-sectional studies (Sullivan et al., 2001; Head et al., 2004; Madden et al., 2004; Sullivan and Pfefferbaum, 2006; Grieve et al., 2007; McLaughlin et al., 2007; Bennett and Madden, 2013). Furthermore, by our longitudinal design and the along-tract analysis we were also able to differentiate such FA changes between tracts and their segments, and between individual participants. Thus, the present longitudinal study provides more detailed information about the aging process of frontal-subcortical WM connectivity than previous cross-sectional reports.

In our study, 19 frontal-subcortical fiber bundles fulfilled our threshold criteria and were selectively detected in each participant of the cohort. A majority of these were fronto-striatal connections. Morphometric changes in fronto-striatal circuits have previously been linked to behavioral differences in normal aging (Head et al., 2004; Ystad et al., 2010; Lockhart et al., 2012), and prefrontal atrophy has been observed in pathological aging (Zhang et al., 2009; Wilson et al., 2012; Jagust, 2013). Our tract-wise FA results demonstrated an average annual reduction of 1.4% across 10 of the identified frontal-subcortical fibers. Moreover, the results provide valuable information about the general rate of FA reduction in normal aging, and (by the along-tract analysis) also the locations of these FA changes. Thus, our study confirms that frontal-subcortical connections and fronto-striatal networks are, to varying degree, vulnerable in normal aging. Generally, the segment-wise FA values along the normalized length of the fibers showed decrease from the first to the second wave, and a further reduction was also revealed at the third time-point in a subset of the participants. As illustrated in the fiber bundle projecting between the caudate and rostral middle frontal region in the right hemisphere, the decrease was most pronounced in the initial (subcortical) segments toward the mid-part of the connection. Detailed spatial analysis of age-related FA change has also been reported by Davis et al. (2009). In their cross-sectional study they found FA decline to increase gradually in the direction from posterior to anterior in the uncinate fasciculus and in the cingulum bundle. Furthermore, their cross-sectional results demonstrated changes in the genu tract (projecting from a seed ROI in the anterior corpus callosum to the target ROI in frontopolar cortex). In our study, we also found significant FA reduction in almost all parts of the ACF. However, our along-tract analyses show that the segment-wise reduction in FA is larger in the initial (posterior) part, both in the ACF, and in several of the investigated frontal-subcortical fiber bundles (as illustrated in Figure 2D and Supplementary Figures 1A,C,E,G). Taken together these cross-sectional and longitudinal results indicate that the amount of age-related FA change is not uniform along the full length of fiber bundles within the frontal region, and that FA decline vary substantially within a tract. Hence, the segment-wise FA profiles contain information beyond the tract-wise mean FA, thus allowing for measuring age effects piece-wise along each fiber.

The major and well-known fiber bundles ACF and CST, were included as comparative tracts for the smaller and less studied frontal-subcortical fiber bundles. For these fiber bundles a fairly strict inclusion criterion was selected, i.e., a minimum of 20 continuous fibers present in at least 90% of the subjects. This criterion may lead to underestimation of the true extent of tract-wise FA changes, but was kept in order to ensure reliable detection of these fiber bundles across the three time-points (cf. Figure 2A). Results from a recent 2-year longitudinal study were not able to detect changes in callosal fibers (Sullivan et al., 2010), whereas our longitudinal results demonstrated a substantial FA decline in the ACF. Several previous cross-sectional studies have reported long association fibers and trans-callosal fibers to be more prone to age-related FA decline compared to projection fibers (Stadlbauer et al., 2008; Voineskos et al., 2012; Bennett and Madden, 2013). Consistent with these findings, our two-wave results revealed a mean annual FA reduction of 1.5% in the ACF, while no significant tract-wise mean FA decline was found in the CST. Moreover, segment-wise FA of the CST showed changes that were much smaller and spatially less consistent than in the along-tract analyses of the ACF. However, a significant but minor FA decline (0.23%) in CST was detected in the left hemisphere when including a third time-point for a subset of the participants. Thus, the current results suggest CST to change at a later age with a more stable FA across time compared to the ACF. These results also correspond to previous findings, as reported by Stadlbauer et al. (2008) and Losnegård et al. (2013), of minor FA changes in the CST. In sum, we might thus speculate if the current data support the retrogenesis hypothesis of WM deterioration, based on its differential maturation dynamics during development (Gogtay et al., 2004; Lebel and Beaulieu, 2011; Lebel et al., 2012). However, evidence for such speculations awaits further investigations. In our study, WM fiber bundles located in the posterior parts of the brain, with different maturation dynamics, were not included.

Regarding mechanistic interpretations, FA is a sensitive marker of WM organization at a microstructural level and can inform us about possible age-related biological processes. FA primarily reflects axonal organization, packing density and membrane integrity and to a smaller extent myelin content (Beaulieu, 2002). Therefore, FA decline can reflect minor alterations in fiber coherence, which can be associated with axonal dystrophy, axonal loss, demyelination, and myelin loss that are characteristic processes of WM aging (Peters, 2002). However, the interpretation of FA in terms of specific neurobiological substrates is currently limited by the resolution of DWI data, and fiber reconstruction approaches. Better differentiation of underlying biological changes may be gained from probabilistic methods and from the inclusion of additional diffusion measures (Burzynska et al., 2010).

Both methodological factors and biological processes may have influenced our results. The frontal-subcortical fiber bundles being studied are smaller, more spread, and less coherent toward the cortical sheet than central parts of the larger fiber pathways, making the first type of fibers more prone to *partial volume effects* (PVE) and faulty registration (Vollmar et al., 2010). Thus, the variable FA changes along the fibers could be linked to locations on the bundle where crossing and branching fibers, high curvature, or intermixing of gray matter occur within the same voxels that contain the tract (Yeatman et al., 2012). In studies of the aging brain, with atrophy of both gray matter and WM (Giorgio et al., 2010; Teipel et al., 2010), this partial volume effect in DWI tractography might be a particular challenge (Alexander et al., 2001; Vos et al., 2011; Metzler-Baddeley et al., 2012). Moreover, our results demonstrate larger variations in segment-wise FA values within the initial (subcortical areas) and toward the terminal parts (frontal areas) of the fibers. This could be caused by registration inaccuracy and the PVE within WM and gray matter boundaries. In this context, the estimated overall tract mean FA, being the most frequently reported tract-wise parameter of WM integrity (Grieve et al., 2007; Kochunov et al., 2007, 2012; Kennedy and Raz, 2009; Teipel et al., 2010; Bennett and Madden, 2013), will certainly be influenced (i.e., summed up) by the localized FA values obtained by an along-tract parameterization. Thus, this detailed analysis can clarify spatial areas along the tract contributing to changes in tract mean FA—contributions either being due to biological processes or to local methodological artifacts. Therefore, further studies are needed to investigate to what extent specific segments along the tracts are more prone to methodological and biological variations than others, and thereby biological interpretations and data quality of FA estimates in tracts and in coarser ROI.

How to distinguish between normal age-related changes vs. pathological neurodegeneration is a challenge in imaging-based investigations of aging (Jagust, 2013). The relevance of our study in this context is the mapping and quantitative change characterization of distinct frontal-subcortical WM connections in single, healthy individuals across three points in time. Our results demonstrated substantial inter-individual variability in the magnitude and rate of FA changes, and with more than 2.0% annual decrease in tract mean FA for some individuals, accentuating what has been detected in previous cross-sectional studies (Sullivan et al., 2001; Madden et al., 2004; Sullivan and Pfefferbaum, 2006; Stadlbauer et al., 2008). In terms of predicting cognitive decline, it is important to consider this generally expected age-related FA reduction, together with a non-negligible individual variability. Thus, the present FA findings may serve as imaging-derived biomarkers for brain connectivity change occurring in the healthy aging brain, and should be taken into consideration in clinical decision making of healthy-aging vs. early signs of neurodegeneration.

Finally, longitudinal imaging studies of healthy aging will be increasingly important in order to better clarify “normal aging trajectories” in space and time in individual brains. Since we will like to compare such “trajectories” also with concomitant behavioral measurements, more powerful statistical methods for longitudinal data analysis (LDA) are called upon. One important class of such LDA methods are linear and non-linear mixed effects models (e.g., Long, 2012), applicable to longitudinal data from cohorts with three or more study waves, enabling estimation of both population fixed effects and individual random effects (Bernal-Rusiel et al., 2012; Reuter et al., 2012). Further research on the current cohort will include investigations of such longitudinal brain-behavior relationships, examining trajectories of both cognitive functions and WM connectivity changes across time using detailed along tract FA analyses and mixed effects models as e.g., in Losnegård et al. (2013) and Sadeghi et al. (2013).

## Author contributions

AV: Statistical analyses and interpretation of the results; writing the manuscript. EH: Design and implementation of workflow and algorithms for image registration, estimation of diffusion tensor, FA, and the along-tract analysis; commenting on the manuscript. MY and JH: Interpretation of the results and critical comments on the manuscript. AJL: PI of the project; design of study and inclusion of participants; writing and commenting on the manuscript. AL: Design of the MRI-protocol and the image analysis approach; writing and commenting on the manuscript. All authors have approved the final version of the manuscript and agreed on all aspects of the work.

### Conflict of interest statement

The authors declare that the research was conducted in the absence of any commercial or financial relationships that could be construed as a potential conflict of interest.

## Supplementary material

Supplementary Figure 1 **Two-wave tract parameterization analysis for the remaining 9 subcortical-frontal fibers where mean FA was substantially reduced. (A)** LH caudate, lateral orbitofrontal; **(B)** RH thalamus, rostral middle frontal; **(C)** RH putamen, lateral orbitofrontal; **(D)** LH caudate, medial orbitofrontal; **(E)** RH caudate, lateral orbitofrontal; **(F)** RH putamen, pars triangularis; **(G)** LH caudate, rostral middle frontal; (H) RH caudate, medial orbitofrontal; (I) RH putamen, lateral orbitofrontal. For more detailed figure description see Figure 2.

Supplementary Figure 2 **Two-wave tract parameterization analysis for the remaining 9 subcortical-frontal fibers where mean FA was preserved. (A)** LH thalamus, superior frontal; **(B)** LH putamen, medial orbitofrontal; **(C)** LH Putamen, rostral middel frontal; **(D)** LH thalamus, rostral middle frontal; **(E)** LH putamen, lateral orbitofrontal; **(F)** LH putamen, lateral orbitofrontal; **(G)** LH caudate, superior frontal; **(H)** LH putamen, pars triangularis; **(I)** LH accumbens, medial orbitofrontal. For more detailed figure description see Figure 2.
