# Supplementary figures and images for: Fractional anisotropy shows differential reduction in frontal-subcortical fiber bundles—A longitudinal MRI study of 76 middle-aged and older adults
Source: Front Aging Neurosci. 2015 May 15;7:81. doi: 10.3389/fnagi.2015.00081 (PMC4432666; doi:10.3389/fnagi.2015.00081)

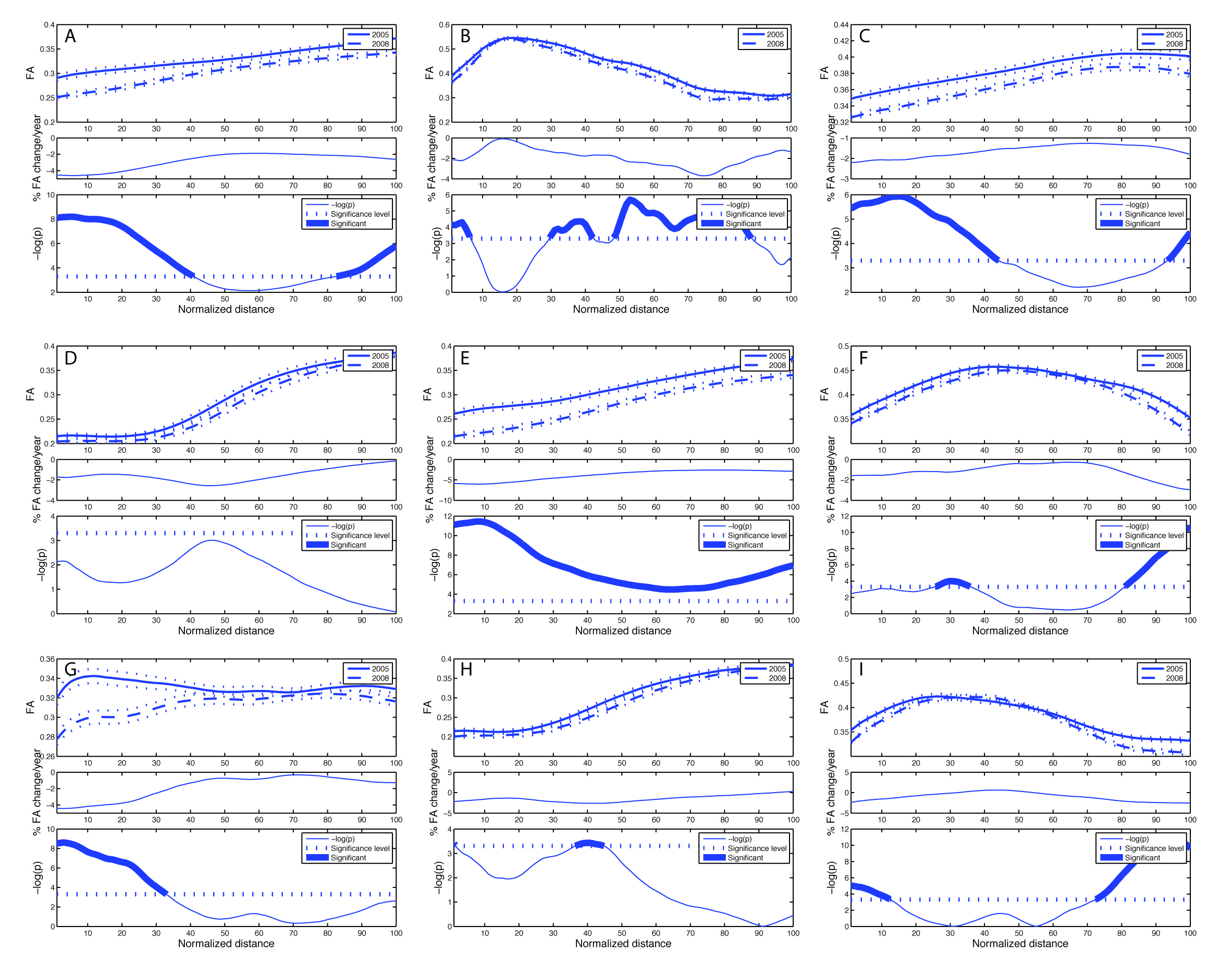

Supplement: Supplementary Figure 1 — Two-wave tract parameterization analysis for the remaining 9 subcortical-frontal fibers where mean FA was substantially reduced. (A) LH caudate, lateral orbitofrontal; (B) RH thalamus, rostral middle frontal; (C) RH putamen, lateral orbitofrontal; (D) LH caudate, medial orbitofrontal; (E) RH caudate, lateral orbitofrontal; (F) RH putamen, pars triangularis; (G) LH caudate, rostral middle frontal; (H) RH caudate, medial orbitofrontal; (I) RH putamen, lateral orbitofrontal. For more detailed figure description see Figure 2. [file Image1.JPEG]

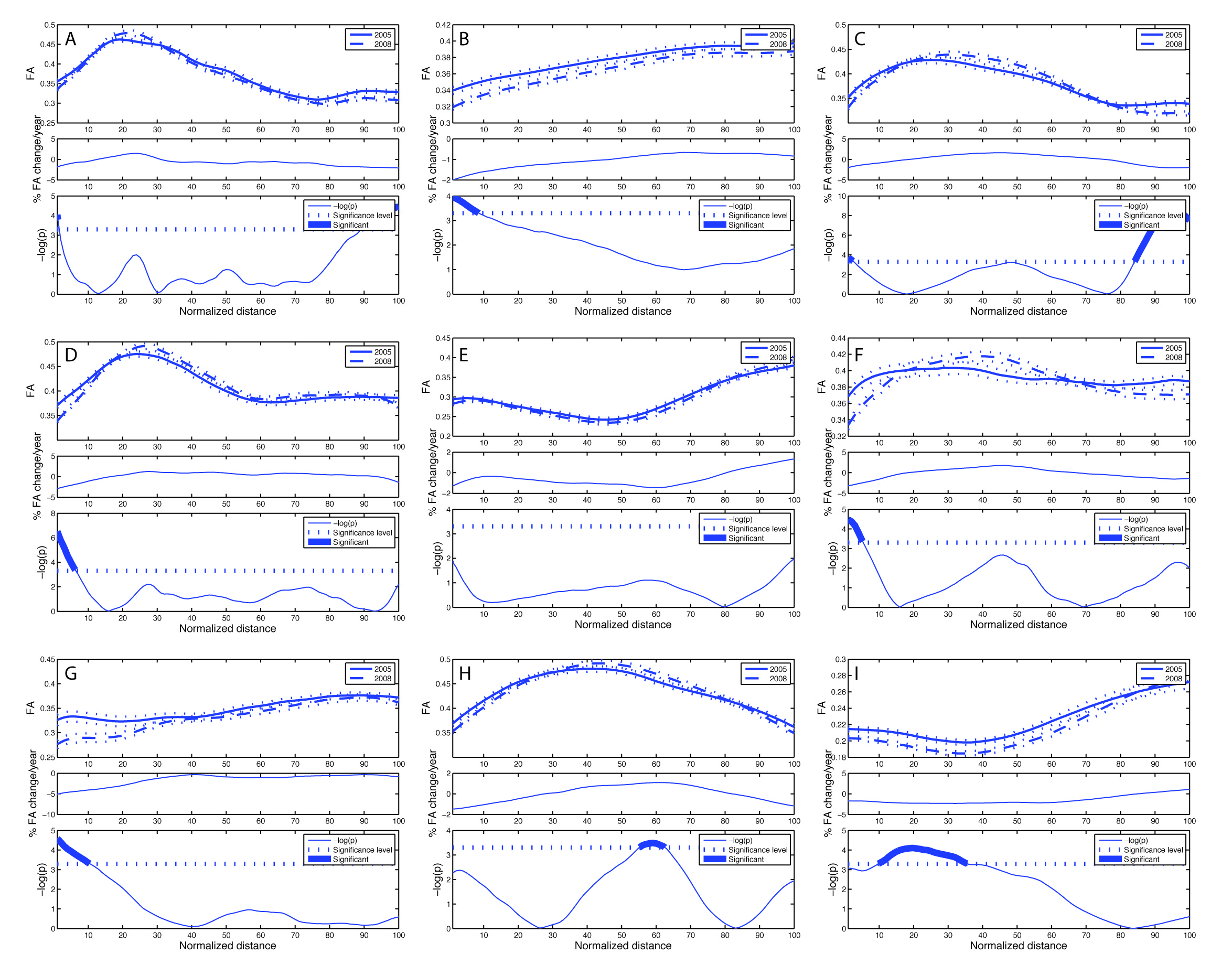

Supplement: Supplementary Figure 2 — Two-wave tract parameterization analysis for the remaining 9 subcortical-frontal fibers where mean FA was preserved. (A) LH thalamus, superior frontal; (B) LH putamen, medial orbitofrontal; (C) LH Putamen, rostral middel frontal; (D) LH thalamus, rostral middle frontal; (E) LH putamen, lateral orbitofrontal; (F) LH putamen, lateral orbitofrontal; (G) LH caudate, superior frontal; (H) LH putamen, pars triangularis; (I) LH accumbens, medial orbitofrontal. For more detailed figure description see Figure 2. [file Image2.JPEG]
